# Critical analysis of pemphigus vulgaris in pregnancy

**DOI:** 10.3389/fimmu.2026.1788979

**Published:** 2026-03-30

**Authors:** Tithi Shah, Alidzhon Baltabaev, Yisong Geng, A. Razzaque Ahmed

**Affiliations:** Department of Dermatology, Tufts University, School of Medicine, Center for Blistering Diseases, Boston, MA, United States

**Keywords:** endocrine changes, hormonal changes, immunological changes, neonatal, pemphigus and pregnancy, pemphigus vulgaris, post-partum, pregnancy

## Abstract

Human pregnancy is a complex, interesting and challenging interplay between the hormonal and immune systems. The presence of an autoimmune disease adds to this complexity. Presence of pemphigus vulgaris (PV) in a pregnant patient should be considered a "high risk pregnancy", by all health care providers involved, from implantation to post-partum care and thereafter. In this systemic review, data from 90 studies involving 111 pemphigus vulgaris patients who were pregnant has been critically analyzed. Patients who had PV before pregnancy and those who developed it during pregnancy were studied and outcomes were compared and are discussed. Systemic corticosteroids (CS) remain the mainstay of treatment. The role of the placenta in producing endogenous cortisol should be considered in adjusting these doses during the last trimester and post-partum. The data in this analysis clearly demonstrates that the clinical and serological control of and the remission of PV is one of the most important factors that influences and predicts maternal health, gestational complications, post-partum exacerbations, neonatal pemphigus and fetal mortality. Observations from this comprehensive review indicate that PV does not preclude successful pregnancy. Critical outcomes measured are probably similar in women who have PV before pregnancy or develop it during pregnancy. The incidence of neonatal pemphigus was 38%, fetal mortality was 9.8% and post-partum flares occurred in 37% of patients. These observations correlated with lack of control of maternal disease during pregnancy. Frequent maternal and fetal monitoring should be considered. Topical therapy should be encouraged since it may decrease the need for higher doses of CS. Azathioprine appears to be the safest immunosuppressive agent in these patients. When available and affordable intravenous immunoglobulin (IVIg) can provide significant benefits. Mucocutaneous disease had more significant consequences than only cutaneous disease. However 70-80% of the patients had oral disease. Therefore oral health care providers should be aware of pemphigus in pregnancy. In some patients, PV may persist after pregnancy, especially if it was present and active post-partum. Patients with PV in a child-bearing age should be advised to get pregnant when PV is in remission. Optimal outcome requires team-work.

## Introduction

Pemphigus vulgaris (PV), a potentially fatal mucocutaneous autoimmune disease is common in patients over 50 years of age. However, in certain regions of the world particularly Middle East and Southeast Asia, PV can occur in young women in the reproductive age group. Considering that PV like other autoimmune disorders is more common in females, it is clinically relevant and beneficial to assess the clinical course of PV during pregnancy. Furthermore, the impact of the pregnancy on the foetus is of significant interest to the parents and to the healthcare professionals.

The purpose of this review was to divide the cohort of patients into two groups: those who developed PV during pregnancy and those who had PV prior to pregnancy. The available data on these two groups was further analysed by the time of onset of disease during the pregnancy. Multiple variables were used to determine the treatment of PV during pregnancy, clinical outcomes of treatment and the postpartum course of PV. Gestational complications, presence of neonatal PV and neonatal mortality were correlated with the course and treatment of PV during pregnancy and other factors which would affect such outcomes. This review facilitates the dermatologists and obstetricians in deciding the best treatment and the most favourable maternal and neonatal outcomes. The cumulative comprehensive data would be beneficial to the patients and their healthcare providers in addressing several issues that relate to this unique and rare clinical situation.

## Material and methods

**Figure 1:**
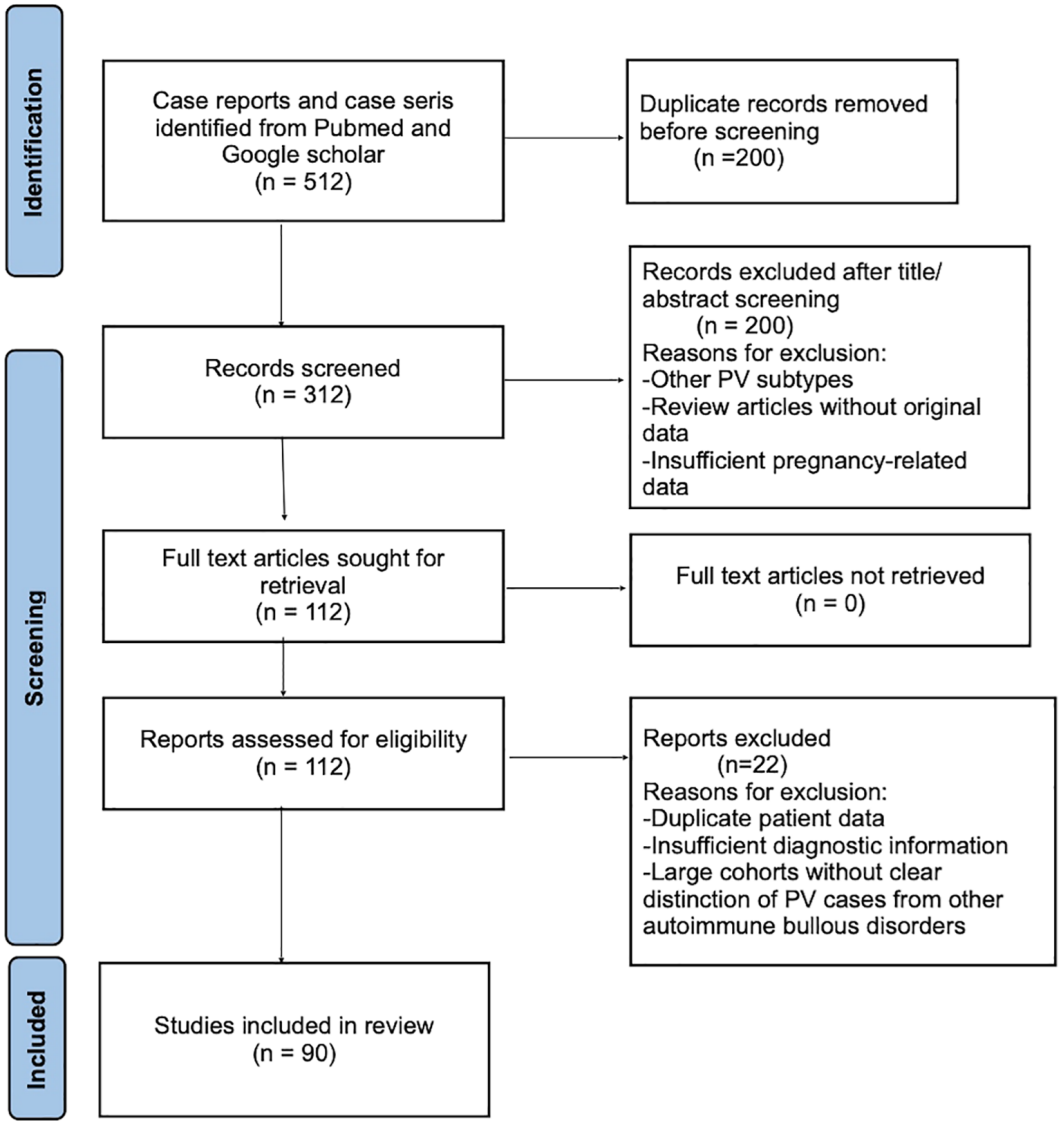
PRISMA 2020 flow diagram.

To identify reported cases of pemphigus vulgaris (PV) occurring prior to or during pregnancy, a systematic literature search was conducted in PubMed and Google Scholar for studies published between 1950 and 2025. The search strategy included combinations of the keywords *“*pemphigus vulgaris,” “pemphigus and pregnancy,” “pregnancy,” “immunological changes,” “endocrine changes,” *and* “hormonal changes.” A PRISMA Flow Diagram (**Figure 1**) documents the identifying screening, eligibility and reasons for including studies. ResearchGate was used solely as a supplementary source to retrieve full-text articles or additional case details when these were not accessible through standard bibliographic databases. Eligible studies included case reports and case series.

The following inclusion criteria were used for data collection: Studies were included if they provided data on diagnosis of pemphigus before or during pregnancy, and provided sufficient clinical information, including onset of PV, clinical outcomes before, during and after pregnancy, treatment used, postpartum outcome, gestational and neonatal complications and neonatal pemphigus.

Following exclusion criteria were used: Studies were excluded if they described other subtypes of pemphigus, lacked sufficient diagnostic information, did not provide extractable pregnancy-specific data for collection, contained duplicate patient reporting, or included clinical trials, guidelines, and review articles in which references to original studies were unavailable.

The patient cohort used in in this analysis consisted of 111 patients reported in 90 studies.

The data on patients was divided into three groups: Group A, consisted of patients who developed PV during pregnancy. When information was provided, it was noted if the pemphigus occurred in the first, second or third trimester. Group B, consisted of patients who had pre-existing PV and became pregnant. In these patients, it was noted if the exacerbations of PV, occurred in the first, second or third trimester. Group C consisted of patients who had PV prior to becoming pregnant, however the authors did not provide information on during exactly which trimester exacerbation occurred.

Groups B and C were combined for purposes of data analysis. Both groups of patients represented patients with pre-existing pemphigus vulgaris prior to pregnancy. Group B and differed only in the availability of the information of which trimester.

The Authors collected data on which trimester of exacerbation to demonstrate that exacerbation can occur at variable intervals. However since patients had pre-existing PV, the time of exacerbation was not consequential, but incidental. It is important to note that clinical characteristics, clinical course, outcomes, complications and treatment outcomes were significantly similar. Hence the observation that the trimester in which the exacerbation occurred did not influence any parameters of our study. Furthermore combining Group B & C facilitate more meaningful statistical analysis, which improved the study quality.

Study selection was performed in accordance with PRISMA 2020 guidelines, with records screened after duplicate removal, full-text articles assessed for eligibility, and included studies summarized in a PRISMA flow diagram (**Figure 1**).

In each of the three groups, following information was noted on each patient.

- Age at onset of PV
- The pregnancy in which PV occurred
- In patients who had PV prior to becoming pregnant, the treatment used to treat PV. The dose of prednisolone used for treatment was divided into high dose prednisolone (>40mg/day) and moderate to low dose (<40mg/day). Furthermore, information on adjunct treatments such as immunosuppressants, rituximab, intravenous immunoglobulin (IVIg) and plasmapheresis, if available, was included.
- Since the time spectrum of data collection was extensive, objective criteria for describing severity were not available for use. Therefore, the clinical profile was described as mucosal only, cutaneous only, or mucocutaneous. The datasets were carefully examined for the presence of oral disease.
- In patients who had PV prior to pregnancy, the clinical outcomes prior to pregnancy.
- Gestational complications
- Treatment during pregnancy was described as high or moderate to low dose prednisolone, pulse therapy and other treatment modalities.
- Based on data provided by authors, the clinical outcome prior to delivery were categorized as, in remission off or on therapy, partial remission, non-responsive to therapy or treatment failures. Non-responsive indicated those patients whose clinical disease did not change despite prolonged systemic therapy.
- Mode of delivery such as normal vaginal or caesarean section
- The clinical course of pemphigus postpartum was noted, especially postpartum exacerbation, its duration and the length of follow up, when provided.
- The following information was noted on neonates: (a) Number of live births (b) Incidence of neonatal complications (c) Incidence and duration of neonatal PV (d) Neonatal mortality (e) Maternal factors and the influence of treatment of PV, on neonate.

### Statistical analysis

For continuous variables, t-tests were used to compare continuous variables between two groups when the data is normally distributed. Wilcox ranked test were used when the data distribution is skewed. Fisher’s exact test was employed to assess the distribution of categorical variables. The p-value of 0.05 was used as the threshold for statistical significance. Because this was an exploratory analysis involving a large number of statistical comparisons, no formal adjustment for multiple testing was performed in order to avoid potential false negative. Results should be interpreted as hypothesis-generating, and p-values near 0.05 may represent type I (false positive) errors. 95% confidence intervals were added. Borderline significance, such as P = .055 were regarded as insignificant. Data analysis was performed using Microsoft Excel and R software.

## Results

### Patient groups

The data base included 111 patients from 90 studies (1–90) presented in **Table 1**.

**Table 1:** Number of patients in each group in this analysis.

	Total	1^st^ trimester	2^nd^ trimester	3^rd^ trimester
Group A (1–37)	41	23	11	7
Group B (1, 11, 38–71)	37	12	18	7
Group C (15, 25, 27, 28, 49, 50, 72^-^90)	33	–	–	–

In group B & C, subsequent pregnancies were reported in four (49, 76, 78, 90) and twin pregnancy was reported in one patient (71).

### Demographics

Age of onset: The mean age of onset in Groups A and B & C was 29.9 years. There was no statistically significant difference in the age of onset between both the groups.

### Gravida

In both groups A and B & C, mean gravida at the time of presentation was two, with no statistically significant difference between the groups, was observed.

Interval between PV and pregnancy.

In group A, onset of PV was at a mean of 14.6 weeks of pregnancy.

In group B & C, the mean interval between the onset of PV and occurrence of pregnancy was 42 months.

### Clinical profile

**Figure 2:**
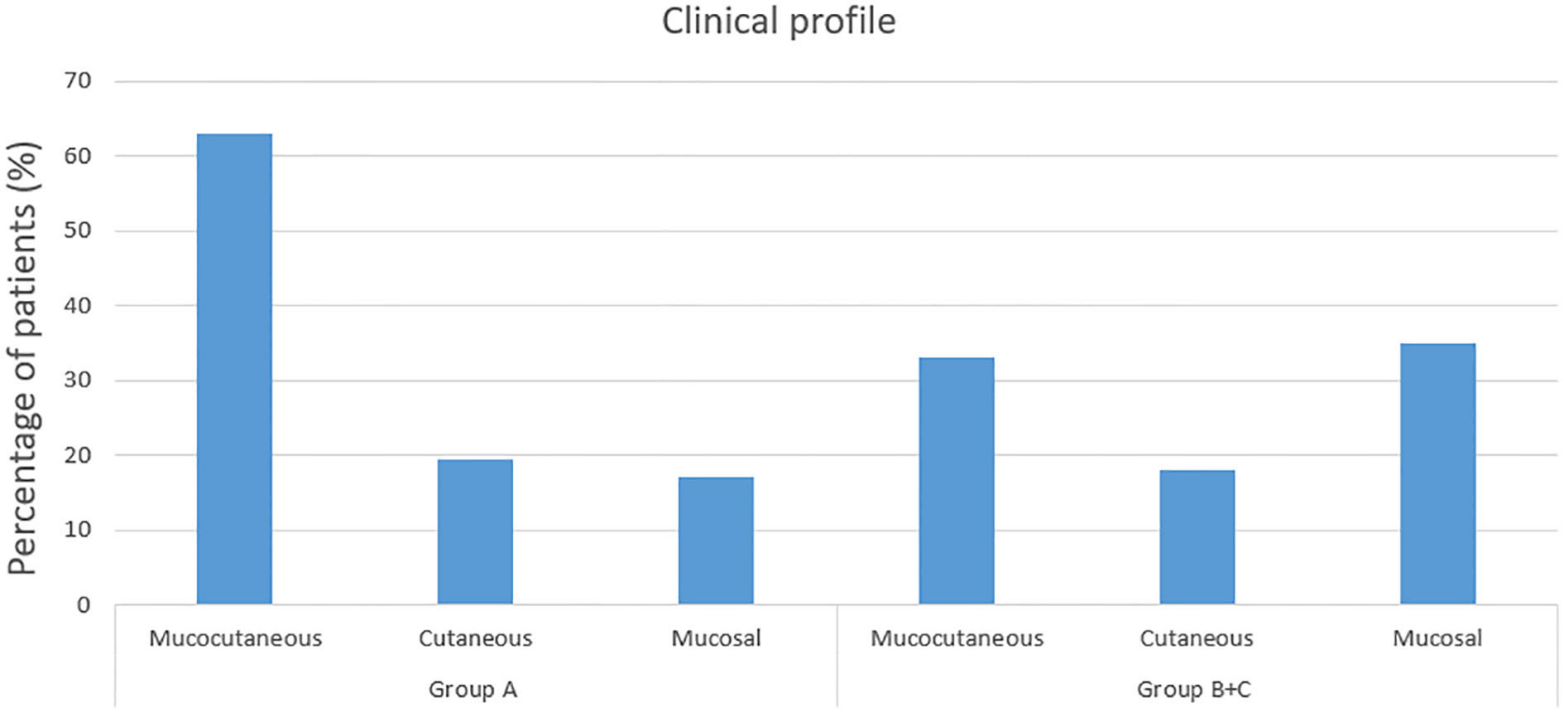
Clinical profile of patients in group A and B+C.

**Figure 3:**
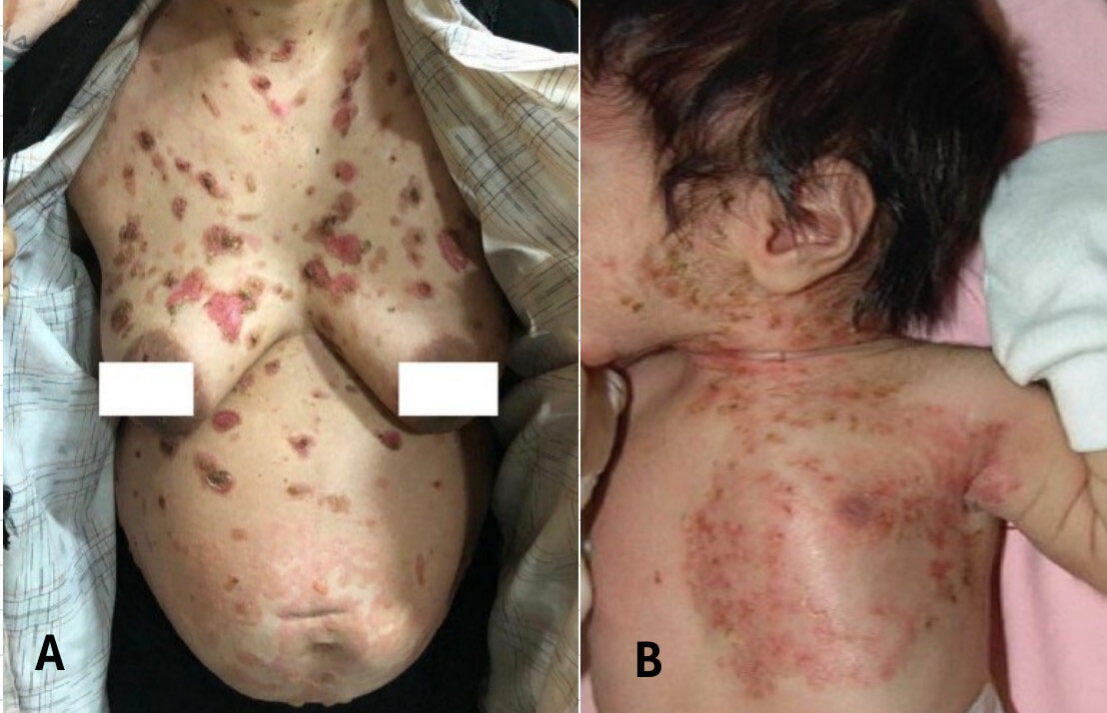
Clinical presentation of pemphigus vulgaris in a pregnant patient **(A)** and neonatal pemphigus **(A, B)** Description of panel **(A)** Reproduced from Mohaghegh et al., 2022 (9) under the Creative Commons Attribution-NonCommercial-NoDerivatives 4.0 International License (CC BY-NC-ND 4.0) (https://creativecommons.org/licenses/by-nc-nd/4.0/). **(B)** Description of panel **(B)** Reproduced from Amer et al., 2007 (90) under the Creative Commons Attribution-NonCommercial-NoDerivatives 4.0 International License (CC BY-NC-ND 4.0) (https://creativecommons.org/licenses/by-nc-nd/4.0/).

Clinical profile of patients in group A and group B & C is represented in **Figures 2A**, **3**.

Mucocutaneous involvement was significantly more frequent in Group A patients (63%) compared to Groups B & C patients (33%), with the difference being statistically significant (OR = 0.292, 95% CI = (0.116, 0.709), p=0.004). The incidence of cutaneous and mucosal lesions was slightly higher in group B & C in comparison to group A, with no statistically significant difference found between the two groups. Oral involvement was more common in Group A patients (80%) compared to Groups B & C patients (68%), with the difference being statistically significant (OR = 0.368, 95% CI = (0.116, 1.047), p=0.044).

### Clinical outcome before pregnancy

Prior to pregnancy, in group B & C, 48% were in complete remission, 29% were in partial remission, 14% were experiencing exacerbation, and 9% had PV diagnosed 1 to 4 months before the onset of pregnancy.

In comparing the duration of presence of pemphigus prior to pregnancy, patients with longer duration (mean: 60.9 months) were in complete remission before pregnancy (11, 25, 28, 39, 41, 44, 46, 47, 49, 50, 53, 58, 62, 64, 74, 78, 79, 81, 86, 89, 90) when compared with patients whose duration of PV prior to pregnancy was shorter (mean: 28.5 months). This difference in duration of disease prior to pregnancy was statistically significant (Wilcoxon rank-sum test: W = 149.5, difference in location = 24.0, 95% CI = (-48.0, -6.0), p = 0.007).

### Treatment before pregnancy

Among patients in group B & C with known clinical outcomes, 54% patients were receiving systemic treatment at the time of pregnancy (11, 27, 38, 41–49, 53, 55, 58, 62, 68, 74, 76, 80–84, 86, 87, 89). High-dose corticosteroids (mean 120 mg) were administered to 8% of patients, experiencing disease exacerbation. Equal number of patients in complete and partial clinical remission received moderate to low dose corticosteroids (mean: 11 mg, 18 mg respectively).

Prior to pregnancy, in addition to corticosteroids, few patients were given adjunct treatments. Azathioprine was administered to 15% of patients in complete remission, 8% in partial remission and 4% with active disease. Furthermore, 4% of patients in partial remission received a combination of rituximab, mycophenolate mofetil (MMF) and intravenous immunoglobulin (IVIg).

There was no statistically significant relation between treatment such as corticosteroids and adjunct treatment given before pregnancy, to clinical outcome before pregnancy, treatment given during pregnancy, gestational complications, postpartum outcome and neonatal outcomes including neonatal complications, neonatal PV and fetal death.

Only in Group B & C, those patients who got adjunct therapy before pregnancy were compared to patients who did not get adjunct therapy prior to pregnancy. The data showed that there was a statistically significant higher incidence of those who got adjunct therapy before pregnancy were most likely going to need adjunct therapy during pregnancy (OR = 5.91, 95% CI = (1.05, 30.33), p=0.022). This could also suggest that severity and extent of disease pre-pregnancy and during pregnancy, did not significantly change in these patients.

### Gestational complications

Gestational complications are presented in **Table 2**. The most frequently observed complications were premature rupture of membranes, preterm birth, and intrauterine growth restriction. No statistically significant difference in the overall incidence of gestational complications was found between the two groups (A vs. B & C; p = 0.683).

**Table 2:** Gestational complications in Group A and B & C.

Group	Incidence	Type of complications
Group A	32%	Premature rupture of membranes (PROM), preterm labor, intrauterine growth retardation (IUGR), meconium stained liquor and unfavourable cervix, oligohydramnios, stillbirth, gestational diabetes, iatrogenic cushing’s syndrome, urinary tract infection, cutaneous herpes simplex infection of lesions
Group B & C	37%	IUGR, placental insufficiency, premature birth, fetal distress, hypertension with tachycardia, PROM, cushing’s syndrome, gestational diabetes, oligohydramnios, fetal growth retardation, stillbirth, gestational thrombocytopenia

Additionally, no statistically significant correlation was found between gestational complications and the treatment given before pregnancy. In correlating treatment given during pregnancy with gestational complications, the data demonstrated that 44% of patients who received any systemic therapy during pregnancy developed at least one complication compared to 21% who received no systemic therapy during pregnancy. This difference was statistically significant (OR = 2.85, 95% CI = 1.14, 7.58), p=0.028). However, no statistically significant association was noted between high dose corticosteroid given during pregnancy and gestational complications.

### Clinical outcome during pregnancy

Clinical outcomes of all patients during pregnancy are presented in **Table 3**.

**Table 3:** Clinical outcome of PV at the end of pregnancy.

	Complete remission	Partial remission	Exacerbation	Non-responders
Group A	28%	50%	19%*	3%
Group B & C	44%	37%	16%	3%

*, Active disease.

No statistically significant difference in clinical outcomes during pregnancy were observed between Group A and Groups B & C.

In patients of Group A, there was a statistically significant shorter duration of PV during pregnancy (mean: 8.6 weeks) in those who achieved complete clinical remission (11, 13, 14, 22, 23, 25, 28, 31, 37) vs those who did not achieve complete clinical remission (mean: 17.9 weeks). This difference was statistically significant (Wilcoxon rank-sum test: W = 147.5, difference in location: 8.0, 95% CI = (2.0, 17.0), p = 0.017).

In contrast, in patients of Group B & C, patients with longer duration of PV prior to pregnancy (mean: 57.9 months) were more likely to achieve complete clinical remission during pregnancy (11, 25, 28, 39, 41, 47–50, 55, 57, 58, 60, 68, 70, 71, 74, 77–79, 81, 86, 89) compared to patients who had shorter duration of PV prior to pregnancy (mean: 30.2 months). This difference was statistically significant (Wilcoxon test: W = 103, difference in location = 23.0, 95% CI = (-3.90, -3.50 x 10^-5^, p = 0.046).

### Treatment during pregnancy

Among patients with known clinical outcomes during pregnancy, 74% in group A and 66% in groups B & C received treatment. Details of these treatments are presented in **Table 4**.

**Table 4:** Treatment during pregnancy and postpartum.

Treatment time	Group	Clinical outcome	Corticosteroids			Adjunct treatments to corticosteroids
			High dose	Moderate-low dose	Dose NA	
During pregnancy	Group A	Complete remission	4% (60 mg)	20% (mean 27 mg)	8%	4%: Plasma exchange
		Partial remission	28% (mean 91 mg)	28% (mean 31 mg)	8%	8%: IvIg 4%: Plasma exchange 4%: Dapsone
		Active disease	4% (300 mg)	–	–	4%: Azathioprine
	Groups B & C	Complete remission	18% (mean 94 mg)	18% (mean 19 mg)	10%	3%: Plasma exchange 3%: IvIg
		Partial remission	15% (mean 95 mg)	18% (mean 12 mg)	5%	5%: Azathioprine
		Exacerbation	5% (108 mg)	8% (mean 20 mg)	3%	–
During postparum	Group A	Complete remission	5% (125 mg)	30% (mean 17 mg)	14%	5%: Azathioprine 5%: IvIg 10%: MMF
		Partial remission	14% (mean 60 mg)	9% (mean 20 mg)	5%	5%: Azathioprine
		Exacerbation	5% (100 mg)	9% (mean 30 mg)	9%	5%: MTX + IvIg 5%: Rituximab 5%:Dapsone
	Groups B & C	Complete remission	6% (50 mg)	29% (mean 14 mg)	6%	6%: Dapsone
		Partial remission	6% (78 mg)	6% (20 mg)	6%	–
		Exacerbation	23% (mean 61 mg)	12% (mean 20 mg)	6%	12%: Azathioprine 6%: Rituximab 6%: Plasma exchange

IVIg, Immunoglobulin; MTX, Methotrexate; MMF, Mycophenolate Mofetil.

There was no statistically significant correlation between the treatment given during pregnancy (corticosteroid and adjuvant treatment) and clinical outcomes during gestation, nor with the systemic corticosteroid dose required in the postpartum period.

### Method of delivery

The incidence of vaginal delivery was 52% in Group A and 67% in Groups B & C. Caesarean delivery was done in 48% and 33% patients of Groups A and B & C respectively. There was no statistically significant difference between the two groups.

### Period of gestation

Group A and group B & C have similar periods of gestation with a mean of 37 weeks.

### Postpartum outcome

Postpartum clinical outcomes in each group are presented in **Table 5**.

**Table 5:** Postpartum clinical outcome of PV patients.

	Complete remission	Partial remission	Exacerbation	Non-responders
Group A	43%	20%	37%	–
Groups B & C	43%	16%	38%*#	3%

*, 1 patient with active disease.

#, 1 patient with postpartum pemphigoid gestationis.

No statistically significant difference in postpartum clinical outcomes was observed between Group A and Groups B & C.

Interestingly, in group B, one patient with pre-existing PV who exacerbated in first trimester, had concomitant PV and pemphigoid gestationis in postpartum period (46).

### Postpartum treatment

Among patients with known clinical outcomes during the postpartum period, 70% in Group A and 45% in Groups B & C received treatment. An additional 17% of patients in Group A and 11% in Groups B & C were initially off all systemic therapy but had relapse of PV and required re-institution of systemic treatment.

A statistically significant high incidence of complete clinical remission in postpartum period (70%) was observed in those who received low doses of corticosteroids in postpartum period (OR = 0.107, 95% CI = 0.014, 0.580), p=0.003). In contrast, the incidence of complete clinical remission in postpartum period in those who received high dose corticosteroids in postpartum period was observed to be 18.8%. Additionally, no significant correlation was identified between the administration of adjunct treatments in the postpartum period with postpartum clinical outcomes.

Treatment details of patients are presented in **Table 4**.

### Postpartum Duration of follow-up

Mean duration of postpartum follow-up was 20 months for Group A and 14 months for Group B & C. There was no statistically significant difference between the two groups.

### Clinical outcome on follow-up

On clinical follow-up, 74% of patients in Group A achieved complete remission, 17% achieved partial remission, and 9% experienced exacerbation. In Groups B & C, 70% achieved complete remission, 22% achieved partial remission and 4% showed no response to treatment. Interestingly, 4% experienced exacerbation of PV. No statistically significant difference was observed during the follow-up period between the clinical outcomes of Groups A and B & C.

### The following observation was made on long term prognosis of PV in the patients

The data indicates that 94.7% of patients who achieved complete clinical remission in postpartum period remained disease free in the long term. While 60% of patients who did not achieve complete clinical remission in postpartum period continued to have PV in the long term follow-up. This difference was statistically significant (OR = 11.42, 95% CI = (1.35, 547.4), p=0.013). Therefore, remission during postpartum period is a significant indicator of long term prognosis of PV in this cohort of patients.

### Neonatal outcomes

In the cohort of 111 pregnancies in the three clinical groups, 102 (92%) live births were reported. The frequency of live birth rate was 90% in Group A and 93% in groups B & C, in those studies that reported such information.

### Neonatal complications and neonatal PV

The incidence and types of neonatal complications, as well as the occurrence of neonatal PV, are summarized in **Table 6**. No statistically significant differences in the incidence of neonatal complications and neonatal PV were observed between the two groups.

**Table 6:** Incidence of neonatal complications and neonatal PV.

Group	Incidence of neonatal complications	Incidence of neonatal PV	Type of complications
Group A	22%	29%	LBW, preterm birth, transient macular rash, cutaneous infection and hypoalbuminemia
Group B & C	24%	44%	Preterm birth, LBW, hyperbilirubinemia, IUGR, meningocele

LBW, Low birth weight.

IUGR, Intrauterine growth retardation.

### Clinical profile of neonates with PV

In the three groups, cutaneous involvement was the predominant manifestation of neonatal PV (75%) (**Figure 3B**), while mucosal and mucocutaneous forms were less frequent. There was no statistically significant difference in the clinical profile of neonates born to mothers in Group A vs Group B & C.

### Clinical outcome of neonatal PV

The mean time to resolution of neonatal PV in both Group A and Groups B & C was two weeks (range: 0.5–6 weeks). Amongst the neonates with neonatal pemphigus, 36% required no treatment. Only 2% received oral prednisone (1 mg/kg for 3 days) (29). The remaining neonates with PV were managed with topical therapies such as steroids, antibiotic creams, emollients, white petroleum jelly. Some patients may have been managed with systemic antibiotics as well.

### Fetal death

Incidence of fetal mortality is high and was reported as 9.8% in Group A and 8.5% in Group B & C. Details on neonatal mortality are presented in **Table 7**. There was no statistically significant difference in the incidence of fetal death between the two groups.

**Table 7:** Fetal death in pregnant pemphigus patients.

Group incidence	No. of cases	Gestational age	Cause of death	Neonatal PV
Group A 9.8% 1^st^ trimester	2	33 weeks, Postpartum Day 2	IUGR, pneumonitis; antenatal death due to marasmus	Yes in one (DIF positive)
2^nd^ trimester	1	25 weeks	CMV pneumonitis (autopsy proven), asymptomatic PV	Yes (DIF positive)
3^rd^ trimester	1	Postpartum Day 10	Macerated skin, neonatal death	Yes (DIF positive)
Group B & C 8.5% 1^st^ trimester	1	27 weeks	Fetal distress	No
2^nd^ trimester	3	28 weeks, 32 weeks, 25 weeks	Neonatal PV, Renal vein thrombosis and interstitial nephritis	Yes in one (DIF positive)
3^rd^ trimester	1	32 weeks	Hepatic congestion, Splenic hypoplasia	Yes (Autopsy finding)
**-**	1	Stillbirth at 36 weeks	–	No

IUGR, Intrauterine growth retardation, CMV, cytomegalovirus, DIF, Direct immunofluorescence.

Patients who achieved complete remission during pregnancy were compared to those who did not achieve complete remission during pregnancy. The incidence of neonatal complications and fetal death was lower in those who achieved complete clinical remission. However, a statistically significant higher incidence was observed for neonatal PV in those who did not achieve clinical remission during pregnancy compared to those who did (OR = 0.290, 95% CI = (0.092, 0.823), p = 0.014).

Adjuvant treatments during pregnancy were associated with a reduced incidence of neonatal PV (OR = 0.310, 95% CI = (0.128, 0.728), p = 0.004) but, paradoxically, correlated with higher rates of fetal death (OR = 0.259, 95% CI = (0.052, 1.031), p = 0.041) and neonatal complications (OR = 3.33, 95% CI = (1.29, 9.26), p = 0.007). A notable correlation was observed between high-dose corticosteroid therapy during pregnancy and neonatal PV, with a markedly lower incidence of neonatal PV (13.8%) in mothers receiving high-dose corticosteroids (OR = 0.259, 95% CI = (0.052, 1.031), p = 0.041), compared to mothers receiving moderate to low dose corticosteroids.

In Group A, in correlating disease clinical distribution with neonatal complications, the data demonstrated that a higher incidence of neonatal complications were observed in mothers with mucocutaneous disease, compared to mothers with only oral or cutaneous disease (OR = 5.27, 95% CI = 0.993, 58.2), p=0.045). While, in Group B & C, a higher incidence of neonatal complications was observed in mothers with mucocutaneous ((OR = 12.1, 95% CI = 3.016, 57.41), p<0.001) and mucosal disease (OR = 0.134, 95% CI = (0.0133, 0.683), p=0.008), compared to mothers with only cutaneous disease.

## Discussion

Pregnancy is a state of immunological tolerance, primarily driven by the interaction between the endocrine system and multiple immune factors. It becomes vital to understand the physiological, hormonal and immunological alterations taking place during a normal pregnancy, in order to study their impact on autoimmune disease. **Table 8** identifies some of the important immune and endocrine changes that take place from implantation through the postpartum period in a normal pregnancy.

**Table 8:** Immune and endocrine changes in normal pregnancy.

	Implantation	1^st^ trimester	2^nd^ trimester	3^rd^ trimester	Postpartum
Treg cells (91, 92)	Increase	Same	Decrease	Decrease	Decrease
T helper cells (91, 93, 94)	Th1 > Th2	Th2 > Th1	Th2 >> Th1	Th2 > Th1 Th1 > Th2 Towards the end of 3^rd^ T	Th1 >> Th2 After 4 weeks postpartum, Th2 returns to pre-pregnancy levels and Th1 predominates.
Th17 (92)	–	Low levels	Increase	Decreases towards the end	Rebound increase
IFN-gamma and TNF-alpha (91, 94–97)	Present in small quantities	Decreased	Decreased	Increases towards the end	Returns to normal by 3 months postpartum
IL-4, IL-5 (91, 94)	–	Increase	Peak levels	Starts decreasing towards the end	Returns to normal by 4 weeks postpartum
B cells (91, 98)	–	Either remains same as pre-pregnancy level or decreases	Either remains same as pre-pregnancy level or decreases	Decreases more towards parturition	Returns to pre-pregnancy levels
Progesterone (99–102)	Levels start increasing since ovulation	Luteal placental shift occurs at around 10 weeks of gestation, following which progesterone increases consistently	Increases	Peak levels	Levels drop sharply
Estrogen (94, 101–103)	Increasing since luteal phase	Increases	Increases	Peak levels	Levels drop sharply
HCG (94, 104)	Increases	Increases, peaks	Declines	Decline	–
Prolactin (94)	–	–	Starts increasing since 2^nd^ trimester	Spike in prolactin	Peak and remains elevated throughout breastfeeding
Cortisol (101, 105)	–	–	–	Peaks in 3^rd^ trimester and during delivery	Starts declining, returning to baseline in 12 weeks postpartum

Progesterone, often referred to as the “pregnancy hormone” (103), together with estrogen, acts at both decidual and peripheral levels to sustain the physiological state of pregnancy. By binding to specific receptors on immune cells and inducing the release of mediators (103), these hormones suppress the Th1-driven pro-inflammatory response while promoting a Th2-dominant anti-inflammatory state. This shift aligns with the pathophysiology of Pemphigus Vulgaris (106). Owing to the similar clinical profile, a bi-directional relationship was observed, with pregnancy inducing and affecting the expression of PV and conversely PV affecting the course and outcome of pregnancy. There is a lack of data in the literature, on immunological and endocrinological changes occurring in pregnant patients with pemphigus vulgaris.

### Pregnancy and postpartum outcome

In this study of pregnancy and pemphigus vulgaris (PV), we have categorized 111 patients into 3 groups (**Table 1**): Group A (41 patients, 40%) included those who developed PV during pregnancy, while Groups B & C (70 patients, 60%) had pre-existing PV. Group B comprised patients with known trimester-specific exacerbation data, and Group C included those without such data. No significant differences in maternal age or gravida were observed between Groups A and B & C, suggesting that demographic factors are unlikely to influence PV onset or outcomes in pregnancy. PV onset was more frequently observed in the first trimester in Group A, similar to previous reports (89, 107), while exacerbations were more commonly noted in the second trimester in Group B. Although these differences did not reach statistical significance in this cohort (p = 0.082), potential mechanisms underlying trimester-specific and postpartum onset and exacerbations are similar to those reported in the literature. In **Box 1** the mechanisms presented by the Authors, are derived from established immunological and endocrine changes described in the literature and provide biological context on how the dynamic immune-endocrine microenvironment of pregnancy may influence disease expression. These mechanisms are not derived from data in this analysis.

Box 1 **Literature- based immunological and endocrine mechanisms potentially contributing to PV in pregnancy and postpartum.**

܀ First trimester: factors contributing to the onset/exacerbation of PV:

1. The luteal placental shift (101): between 7–10 weeks of gestation, progesterone production is transferred from corpus luteum to placenta, resulting in a temporary progesterone decrease before increasing, once the placenta takes over. This shift may influence the onset/exacerbation of PV as placental endocrine factors have a strong influence on maternal immune system

**2.** Fetal-maternal immune interaction: Between 8–12 weeks of gestation, maternal peripheral blood comes into close contact with fetal trophoblast cells (108), which can modulate the maternal immune system and potentially influence the development of autoimmune conditions like PV.

3. Transition from Th1 to Th2 immune response: The 1st trimester marks the initiation of the transition from a Th1-dominant to a Th2-dominant immune response. This shift could play a role in the enhanced susceptibility to PV.

**Figure 4:**
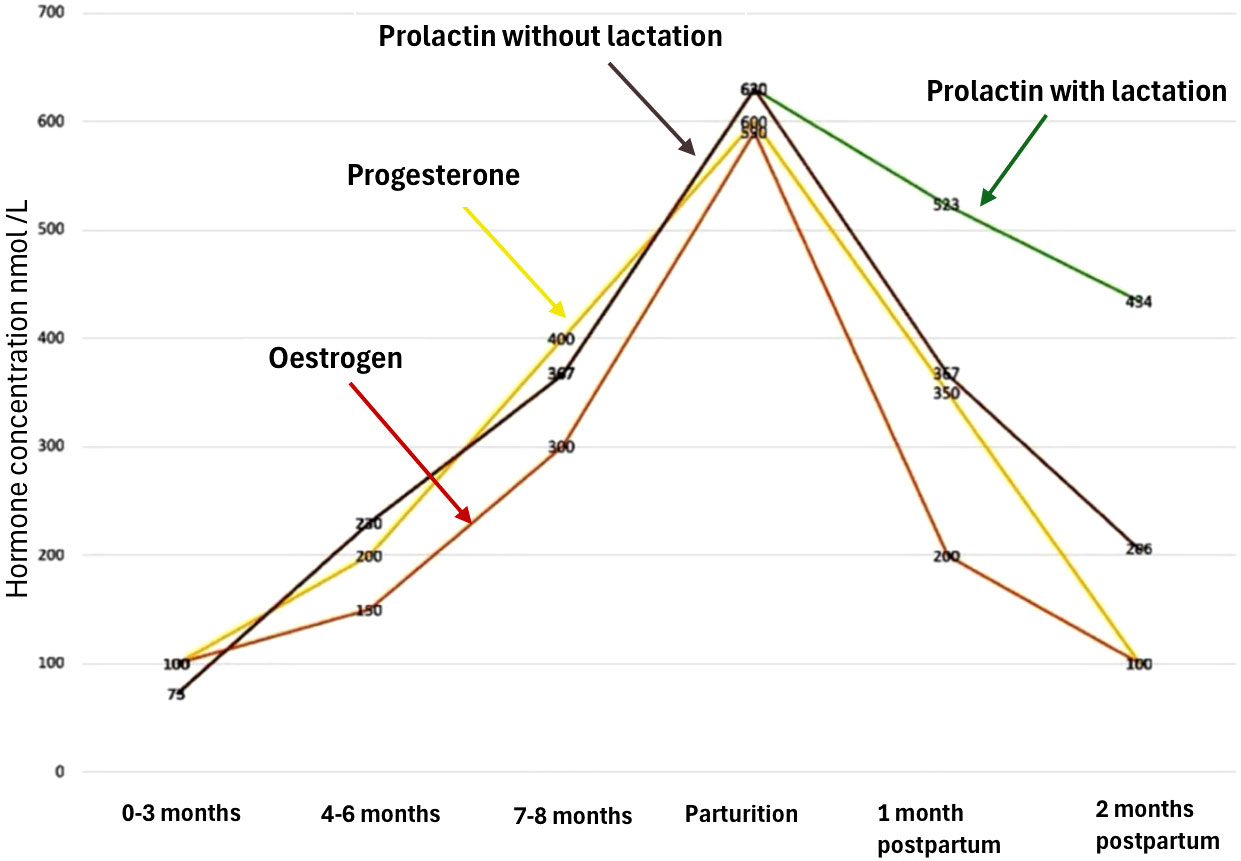
Hormonal changes during pregnancy and postpartum. Reproduced from Kodogo et al., 2019 (111), *Clinical Research in Cardiology*, with permission from the publisher.

܀ Second trimester is characterized by a predominant anti-inflammatory immune milieu (109), further influenced by the use of corticosteroids in many patients with pre-existing PV. Nevertheless, several factors may contribute to the onset or exacerbation of PV during this period:

1. Peak Th2 immune response: Th2 cytokines such as IL-4, IL-5, and IL-10 are at their peak in 2^nd^ trimester (94), suggesting peak Th2 response which in turn can aggravate PV.

2. Elevated Th17 levels: Few studies suggest a role of Th17 cells in PV (110) and from 2^nd^ trimester to postpartum there is an increase in Th17/Treg cell ratio (108).

3. Rising levels of progesterone, oestrogen (**Figure 4**)

܀ Third trimester: The lowest incidence of occurence and exacerbation of PV, are observed during the third trimester (**Table 1**). This can be due to high levels of endogenous cortisol levels produced by placenta along with elevated progesterone levels, leading to significant immunosuppression (89, 105).

܀ Postpartum exacerbation of PV is frequently observed, due to the rapid decrease and eventual loss of systemic corticosteroids which were secreted by the placenta prior to delivery, after the loss of placenta (89).

At the time of presentation, Group A patients demonstrated a broader disease profile, with significantly higher rates of mucocutaneous, cutaneous, and oral involvement compared with Group B. This suggests that pregnancy-onset PV may present with more extensive disease involvement, necessitating close monitoring and early intervention.

In Group B & C patients, a longer duration of disease stabilization prior to conception was associated with a greater likelihood of remission before pregnancy, whereas corticosteroid dose and adjunct therapies did not influence outcomes. This indicates that achieving and maintaining remission for a considerable period before conception is more critical for maternal outcomes than type or dose of systemic corticosteroids.

During pregnancy, in Group A patients, shorter duration of PV was significantly associated with clinical remission. In contrast, in Group B & C patients, longer duration of PV prior to pregnancy was significantly associated with clinical remission. These findings highlight the importance of early diagnosis and prompt treatment in new-onset PV during pregnancy, while in women with pre-existing PV, sustained remission for a considerable period prior to conception appears to be critical for favourable outcomes.

Although corticosteroid dose during pregnancy was not associated with clinical outcomes, the use of high-dose corticosteroids was significantly linked to gestational complications. This likely reflects the fact that women with more severe PV required higher doses and subsequently developed complications. Furthermore, in Group B & C, patients who received adjunct therapy before pregnancy were more likely to require adjunct treatment during pregnancy. This suggests that the factor(s) that necessitated the need for adjunct therapy before pregnancy were present during pregnancy. These observations underscore the need for careful dose balancing, aiming for disease control with the lowest effective regimen to minimize maternal and obstetric risks.

Postpartum outcomes in our study revealed no significant differences between Groups A and B & C, with similar rates of exacerbation observed in both groups (37%).

In our cohort, corticosteroid dose emerged as a key determinant of postpartum outcomes, with moderate-to-low dosing associated with higher rates of complete remission. While deciding on the dose of corticosteroid to use in a pregnant patient with pemphigus, the treating Dermatologist needs to consider that the placenta also secretes corticosteroid hormones (89). As pregnancy progresses, rising endogenous cortisol levels may contribute to relative immunosuppression in late gestation (89). From a practical standpoint, cautious tapering of exogenous glucocorticoids may be considered in clinically stable patients during the third trimester, provided close monitoring is ensured. However, following delivery, the abrupt decline in placental hormones and endogenous cortisol may predispose to disease exacerbation (89). Anticipating this physiological shift, a temporary adjustment or escalation of systemic corticosteroids immediately postpartum may help prevent flares. This strategy should be individualized, particularly in breastfeeding mothers, as glucocorticoids are excreted in breast milk (106) and unnecessary high dosing should be avoided. Mothers must wait for minimum four hours between taking prednisone and breast feeding, for neonatal and infant safety.

Importantly, postpartum remission status was strongly predictive of long-term prognosis, as 94.7% of women in remission at postpartum maintained remission at follow-up compared to 60.0% of those who did not achieve remission prior to delivery. These results emphasize the postpartum period as a critical window for disease monitoring and therapeutic optimization, as outcomes during this stage strongly shape the long-term clinical trajectory.

### Neonatal outcome

In normal pregnancy, despite considerable changes in maternal immune system, fetus is not rejected due to several protective mechanisms:

- Major histocompatibility complex (MHC) antigens: Trophoblast cells lack MHC class I and II antigens and instead express non-classical antigens including HLA-G, which protects the placenta and fetus from maternal T cell and uterine natural killer cell attack (112).
- Th2 polarized immune response
- Endocrine factors: Human chorionic gonadotropin (HCG), progresterone, estrogen play an important role in modulating immune response and establishing immune tolerance.

In pregnant patients with PV, neonatal outcomes can range from normal live births to neonatal complications like preterm birth and IUGR, neonatal pemphigus vulgaris (NPV), stillbirth, or fetal death. Poor neonatal outcomes are considered to be associated with severity of maternal disease, high titre of maternal autoantibodies such as indirect immunofluorescence autoantibody titre of 1:160 (113), aggressive systemic therapy particularly with high dose corticosteroids and in some cases, concurrent viral infections of the maternal genital tract.

Neonatal PV is widely attributed to the transplacental transfer of maternal anti-desmoglein 3 IgG4 autoantibodies, as described in previous studies (113, 114). However, its occurrence is multifactorial and is not solely dependent on active maternal disease or elevated autoantibody titers (114). Neonatal skin contains both, desmoglein-1 and desmoglein-3, throughout the epidermis, whereas in the adult skin, Dsg-1 is confined to the upper layers and Dsg-3 is predominantly localized to the lower layers of the epidermis and mucosal epithelium (113). As a result, maternal autoantibodies against Dsg-1 and Dsg-3 in neonatal PV typically manifests with extensive blistering of the skin and minimal mucosal lesions (89, 114), consistent with observations in our study.

In our cohort, the overall incidence of Neonatal PV was 38%, with a slightly high incidence in Group B & C (44% vs 29% in Group A), although this difference was not statistically significant (p=0.160). Neonatal PV undergoes self-resolution within 2–3 weeks, as seen in this cohort and does not have any long-term consequence in neonates (115).

In our cohort, stillbirth is reported in 8 patients (7%) and postnatal death in 2 patients (1.7%) (**Table 7**). Neonatal PV was diagnosed in 5 of these 10 patients (50%), either through direct immunoflourecnce testing or biopsy during autopsy, suggesting the potential role neonatal PV might have in stillbirth.

Importantly, maternal disease control strongly influenced neonatal outcomes. Neonates born to mothers in complete remission during pregnancy had significantly lower rates of neonatal PV (p = 0.014). Maternal therapy also played a role: high-dose corticosteroid use during pregnancy was associated with markedly lower neonatal PV incidence (13.8%, p = 0.04), and adjunct treatments reduced neonatal PV (p = 0.004), although they correlated with higher rates of fetal death (p = 0.041) and neonatal complications (p = 0.007). Furthermore, maternal mucocutaneous lesions were significantly associated with increased neonatal complications in both Group A (p = 0.045) and Groups B & C (p < 0.001), while maternal mucosal lesions predicted neonatal complications in Group B + C (p = 0.008).

Collectively, these observations demonstrate that while neonatal PV is typically transient and self-resolving, maternal disease activity and treatment patterns exert a major influence on neonatal outcomes. Achieving maternal remission during pregnancy appears to be the most effective strategy to reduce neonatal PV. While careful optimization of therapy, particularly corticosteroid dosing, it is essential to balance maternal disease control with fetal safety.

Treatment of pemphigus vulgaris during pregnancy and lactation.

Systemic corticosteroids, particularly prednisone, remain the first-line treatment for pemphigus vulgaris in pregnancy (107). In our cohort, no statistically significant differences were found between high-dose and moderate-to-low corticosteroid regimens given before, during, or after pregnancy, nor between corticosteroid dose and clinical outcomes before and during pregnancy. However, high-dose corticosteroid use during pregnancy was positively correlated with increased rates of gestational and neonatal complications and fetal death, while being associated with a lower incidence of neonatal PV. These findings underscore the importance of customizing corticosteroid regimens to disease severity while prioritizing the lowest effective dose, according to the current clinical recommendations (115). Treatment approaches did not differ between patients with pre-existing PV and those developing PV during pregnancy (115).

High-dose corticosteroids should be avoided around conception, as they may interfere with the peri-implantation inflammatory response required for normal implantation, placentation, and fetal growth (116). In addition, certain immunosuppressive therapies must be discontinued prior to planned conception: mycophenolate mofetil at least 6 weeks before, cyclophosphamide and methotrexate 3–6 months before, and rituximab at least 12 months before pregnancy (117). Azathioprine, which is category D in pregnancy, according to FDA, may be considered as a second-line drug for women unresponsive to prednisone alone (106). Other therapeutic options considered relatively safe in pregnancy include dapsone, plasmapheresis, and intravenous immunoglobulin (IVIg), with topical or intralesional corticosteroids used as adjuncts to systemic therapy (106, 118).

In our cohort, IVIg was administered as part of combination therapy to one patient before pregnancy and one during pregnancy in Groups B + C, and to two patients during and two patients after pregnancy in Group A. All five pregnancies proceeded without complications, there were no postpartum exacerbations, and all neonates were healthy. These findings are consistent with those reported by Ahmed et al. (119), suggesting that IVIg is a safe and effective therapy during pregnancy.

During our analysis of 111 patients, in these patients who developed PV during pregnancy, we observed that few patients had not received any systemic treatment. In patients who received no treatment, 21% developed gestational complications, postpartum exacerbations occurred in nearly 30% and neonatal PV developed in approximately 50% of neonates. This observation emphasizes the importance of maintaining disease control during pregnancy to reduce adverse maternal and neonatal outcomes.

For women newly diagnosed with PV who desire future pregnancy, achieving sustained disease control prior to conception is strongly advisable. Based on our observations, authors suggest that systemic corticosteroids should remain the mainstay of therapy to control active disease. Intensive disease management should be undertaken before conception is planned, with the goal of achieving clinical remission or stable low disease activity on the lowest effective corticosteroid dose. Azathioprine may be introduced as a steroid-sparing agent in patients requiring additional immunosuppression and, if effective and well tolerated, may be continued throughout pregnancy.

Other adjunct immunosuppressive agents may be required in selected patients with severe or refractory disease; however, such agents should be discontinued prior to planned conception according to the recommended washout periods discussed above.

It would be worth suggesting, that in situations where use of IVIg is possible, IVIg may be used initially, as first line therapy to treatment of PV in a pregnant patient. If and when, IVIg cannot completely control PV, then addition of systemic corticosteroids with or without adjunct therapies can be easily undertaken. The possible benefit could be that the simultaneous use of IVIg would lower the dose of systemic corticosteroids that may be needed. The transplacental transfer of IgG to the fetus, could potentially benefit fetal health.

For mothers with PV who are breastfeeding, treatment options should prioritize neonatal safety such as avoiding application of high-potency topical corticosteroids directly to the nipple. Systemic corticosteroids should be ideally administered four hours before breastfeeding. Azathioprine, IVIg, and dapsone are considered safe. In contrast, cyclophosphamide, rituximab, methotrexate, and mycophenolate mofetil should be avoided due to the risk of transfer into breast milk (106, 118).

The observations made from this comprehensive review indicate that pemphigus vulgaris does not preclude successful pregnancy, and that clinical outcomes are broadly similar in women who develop the disease during pregnancy and those with pre-existing disease. Available evidence does not demonstrate consistent pregnancy-specific alterations in endocrine or immune pathways beyond normal gestational physiology. It is important to note that the placenta represents a critical endogenous source of glucocorticoids during pregnancy, underscoring the need for careful clinical judgment when determining systemic corticosteroid dosing in pregnant patients with pemphigus vulgaris. The majority of neonates reported in the literature were healthy, while gestational and neonatal complications were primarily associated with maternal disease activity and treatment used. Although neonatal pemphigus is typically transient, it can be severe and, in rare cases, fatal. Achieving and maintaining maternal disease remission during pregnancy remains the most effective strategy to minimize maternal morbidity and reduce the risk of adverse neonatal outcomes.

The authors have chosen three reviews, including the ones by Lan et al, 2024 (107) and Lin et al, 2015 (113). There were significant differences between the other reviews. Instead of highlighting them, the authors have described the important aspects of these reviews. They are as follows:

In a review of PV during pregnancy by Lan et al. (107) from China, 57 patients from 42 studies were studied. Patients had PV before and during pregnancy but 7% developed PV only post-partum. Interestingly, 81.5% responded to systemic corticosteroids (CS) but surprisingly 16% of the patients failed to respond to CS and needed alternate treatments. In the India cohort, 37% of neonates had neonatal pemphigus. However, neonatal mortality was not reported by them. Six printed pages contained raw data from their 57 patients in tabular form. This impressive data set has not been published by any other group.

In a review from India, De et al. (113) published data from 83 studies reported between 1950 and 2023. However, the exact number of patients studied was not mentioned. It was not always clear which observations or conclusions made were derived from exactly which data set. The review was robust in providing information on the interaction between the immune and endocrine systems, maternal and fetal risks, drug management, and the broad aspect of the physiology of normal pregnancy and its possible interactions with pemphigus, though specific studies were not cited.

Oral medicine specialists from China, Lin et al. (115), analysed 47 cases of pemphigus and pregnancy reported between 1966 and 2014. Among these, 71.4% had PV, 18% had PF, and, interestingly, 4.8% had Pemphigus Vegetans. While, 57.1% of the neonates in their cohort had pemphigus, 38.1% had PV, and surprisingly, 19% had PF. Post-partum exacerbation of pemphigus was observed in 30.8% of patients. In their publications, the authors chose to emphasize the clinical outcomes in neonates.

It appears that different authors focused on different aspects of this subject, based on what they considered important or relevant. Consequently, each had a different database. Each review provided important, useful information, but often differed in content, in some aspects.

The current review uses the largest database ever reported. Consequently, it covers every clinically relevant aspect of the total subject matter. The purpose of this wide spectrum of information was to provide the readership a comprehensive overview. Most importantly, it provides future authors a framework to do their research, as advances in science and medicine, influence this specific area of clinical practice.

Nonetheless, the findings of the present review are broadly consistent with previously published systematic reviews. Lin et al. reported postpartum exacerbation rates of 30.8% in patients with pregnancy-onset PV and 9.5% in those with pre-existing disease (115); in contrast, we observed comparable postpartum exacerbation rates in both groups (37% in Group A and 38% in Groups B& C), suggesting that the postpartum period itself represents a shared high-risk phase regardless of disease onset timing. The reported incidence of neonatal pemphigus vulgaris (PV) in the literature ranges from 1.4% to 37%, as summarized in prior reviews including that reported by Lan et al. (89, 107, 115). In our cohort, neonatal PV occurred in 38% of neonates, marginally exceeding previously reported rates. The overall fetal mortality rate observed in this study was 9%. Previously reported rates in the literature ranged from 1.4% to 27% (113, 115). Importantly, beyond confirming many of these aggregate outcomes, this review identifies clinically relevant determinants not systematically evaluated in prior reviews, including the influence of maternal remission status during pregnancy, duration of disease stabilization before conception, corticosteroid dosing, use of adjunct therapy, and influence of the extent of mucocutaneous involvement of maternal disease on neonatal outcomes.

### Limitations

This study has certain limitations that should be considered when interpreting the findings. The data were derived exclusively from published case reports and case series spanning several decades, which inherently introduces heterogeneity in diagnostic criteria, treatment approaches, follow-up duration, and reporting standards. Given the retrospective and descriptive nature of the available literature, standardized measures of disease severity and uniform outcome definitions were not consistently available, limiting the ability to perform more robust comparative analyses. Additionally, incomplete reporting in some cases, particularly regarding trimester-specific exacerbations, treatment dosing details, and long-term neonatal follow-up, may have influenced subgroup analyses. The possibility of publication bias must also be acknowledged, as more severe or unusual cases were more likely to be reported. From a scientific perspective, the most serious limitation is that in 7.5 decades, no serious studies on investigations have been done in pregnant pemphigus patients to study hormonal changes or have performed immunological studies to better understand the influence the autoimmunity on both. Finally, although statistical tests were applied, the exploratory nature of the analysis, multiple comparisons, and the relatively small sample sizes in certain subgroups, limited definitive causal inferences.

Suggestion for patients newly diagnosed with pemphigus and pregnancy irrespective of whether pemphigus was present before pregnancy or occurred during pregnancy, both clinical situations should be considered as “high risk pregnancies”, by health care professionals involved in the care of these patients. The Authors suggest that there should be careful maternal and fetal monitoring to secure a desirable and optimal outcome. Patients who had pre-existing pemphigus and become unexpectedly pregnant, health care providers, dermatologists and their obstetricians need to provide some insights that may help patients better cope and manage their disease, during their pregnancy.

Patients should be clearly warned not to discontinue their existing therapies, especially systemic corticosteroids, once they discover they are pregnant.

The second most important step is detailed clinical evaluation of the extent and severity of the disease. Severe disease, with multiple sites of involvement, can have a more difficult clinical course and greater likelihood of neonatal emphigus. Oral pharyngeal or esophageal involvement can cause difficulty eating or swallowing. Such patients need advice by a dietician so that protein and caloric intake is adequate for a pregnant mother. Serum protein and albumin levels should be checked periodically. If facilities are available, checking and following titers of pemphigus specific autoantibodies, would be prudent. Attempts should be made to reduce, and possibly eliminate serum autoantibodies before delivery. Authors have suggested that pemphigus autoantibodies at levels 1:160 or higher by indirect immunofluorescent can carry a more serious prognosis (113).

Use of aggressive topical therapy expedites healing, and can be help in reducing systemic corticosteroid dose. Since there is a natural state of immune suppression, which is compounded by systemic corticosteroid therapy, these patients are highly susceptible to infection. Hence cultures with antibiotic sensitivity should be performed from multiple sites to avoid systemic infection. Topical skin care consists of soaks to remove skin debris, scabs and scales, followed by application of topical corticosteroid creams. If systemic CS are used, very high potency creams can be avoided and may not be necessary. Patients should be repeatedly reminded that clinical and possibly serological control of pemphigus early in pregnancy, maybe the safest method to ensure an uneventful pregnancy and healthy neonate. Higher doses of prednisone maybe given in the first and second trimester, reduced in the third trimester and increased again in the post-partum period, to follow intrinsic cortisol production during pregnancy and post-partum.

Intravenous immunoglobulin (IVIg) has shown to be an important therapeutic agent in the management of pregnancy in patients with pemphigus, other autoimmune blistering diseases and several systemic autoimmune diseases affecting different organ systems. A major limiting factor that prohibits its use is its high cost. However, in countries where the State Health Care Systems or private insurances will cover the cost, it should be used particularly in certain special circumstances. Its multiple benefits have been demonstrated in patients with pemphigus during the course of their pregnancy and the post-partum period (2, 119). Multiple studies have shown that IVIg plays a critical role in the management of pemphigoid gestationis, another antibody mediated autoimmune blistering disease (120–123). It is particularly useful in patients who are refractory to conventional therapies and during the post-partum period. Most investigators have reported good clinical outcomes.

IVIg has been a valuable therapeutic agent in treating patients with many other autoimmune diseases involving multiple organ systems especially lupus, multiple sclerosis, immune thrombocytopenic purpura among others (124–131). Its beneficial effects have been reported during the pregnancy and the post-partum period. The. Collective data from these multiple sources, would suggest that certain generalizations can be made and these observations could facilitate clinician’s in making certain applications. The data would also indicate that IVIg would be of significant benefit in treating those pregnant patients with autoimmune diseases, who cannot tolerate conventional therapies or develop significant side effects that can potentially harm the mother and/or the fetus.

These observations suggest that IVIg can have the following beneficial effects in these pregnant patients. It can produce rapid clinical recovery, maintains clinical remission and prevents post-partum flare. In patients with pemphigus, it has the potential to prevent neonatal pemphigus. It has the much needed potential to produce serological remission before delivery. In complex cases with multiple comorbidities and a high-risk pregnancy, it would be reasonably advisable to attempt using IVIg as monotherapy. Several studies have documented its steroid sparing effect. The recommended dose and protocol in most studies is 2gms/kg/cycle, every month during pregnancy and at least four months post-partum.

Patients who have been pregnant once with pemphigus and want to have a second pregnancy should receive serious counselling regarding contraception and birth control. They should be advised to remain drug and disease free for minimally six months, before planning a pregnancy.

Suggestion to a patient who develops pemphigus while already pregnant will be similar. However the most important concerns would be confirming the diagnosis by immunopathology and determining titers of autoantibody. Such pregnancies are high risk and should have close maternal and fetal monitoring. Aggressive topical therapy is warranted and systemic corticosteroid doses carefully administered and adjusted based on clinical severity, clinical response and clinical course. Every attempt should be made to control clinical disease as soon as possible and prior to delivery.

The data in the literature on post-partum follow-up is limited. Patients should be on long-term follow-up to ensure that pemphigus does not recur and serological remission is also maintained. This should be the responsibility of the consulting dermatologist.

Nevertheless, despite these constraints, and acknowledged limitations, this study provides one of the most comprehensive syntheses of data available on pemphigus vulgaris in pregnancy to date. It offers clinically meaningful insights to health care providers and healthcare educators involved in the maternal and neonatal management of these patients.

## Data Availability

The original contributions presented in the study are included in the article/supplementary material, further inquiries can be directed to the corresponding author/s.
